# Supplementary material for: Complex‐centric proteome profiling by SEC‐SWATH‐MS
Source: Mol Syst Biol. 2019 Jan 14;15(1):e8438. doi: 10.15252/msb.20188438 (PMC6346213; doi:10.15252/msb.20188438)
Supplement: Supplementary file 7 — Dataset EV6 [file MSB-15-e8438-s007.zip › feature_plots_bioplex/Q6P2Q9.pdf]

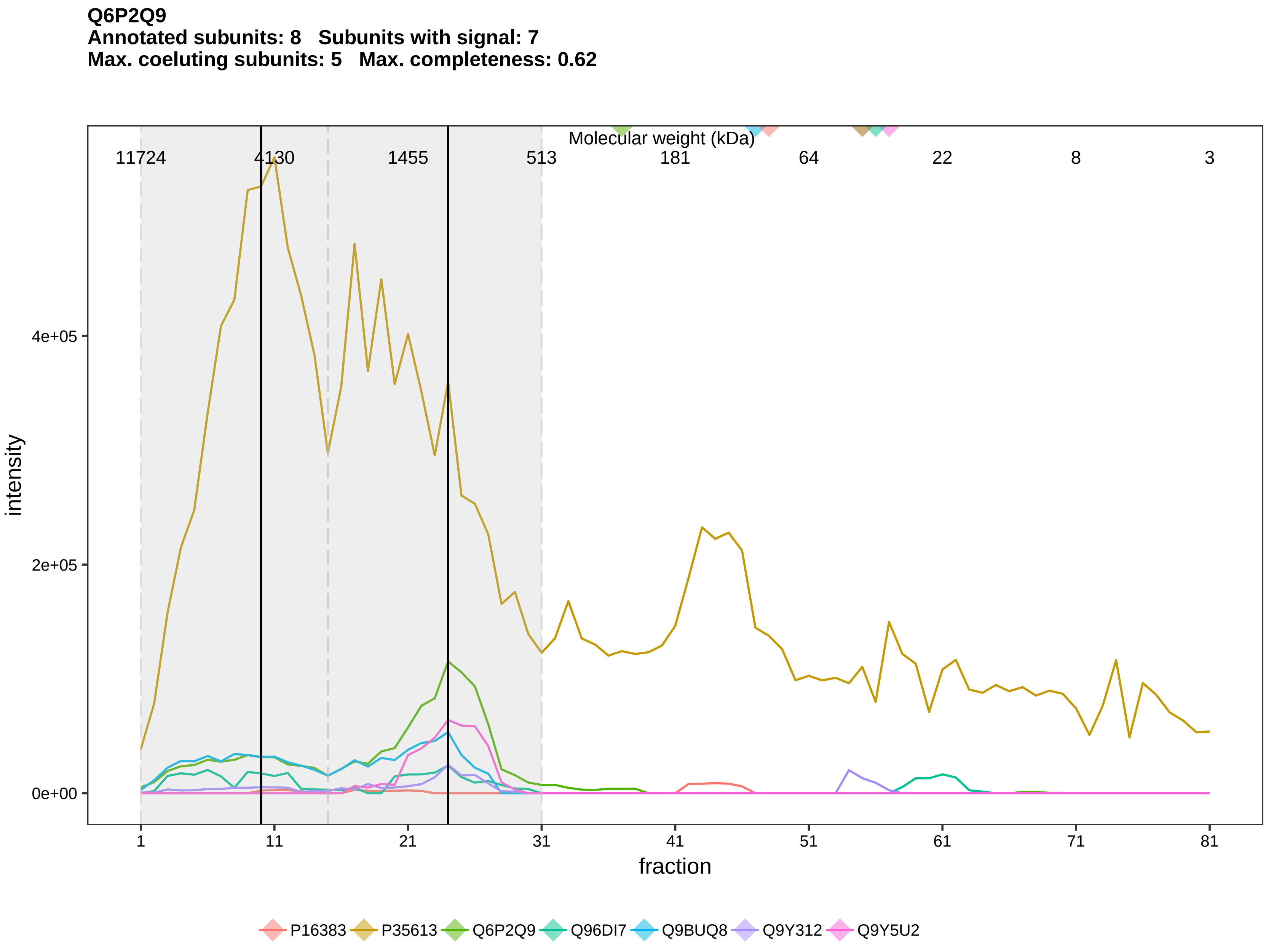
Q6P2Q9
Annotated subunits: 8 Subunits with signal: 7
Max. coeluting subunits: 5 Max. completeness: 0.62
Molecular weight (kDa)
11724
4130
1455
513
181
64
22
8
3
intensity
4e+05
2e+05
0e+00
1
11
21
31
41
51
61
71
81
fraction
P16383
P35613
Q6P2Q9
Q96DI7
Q9BUQ8
Q9Y312
Q9Y5U2
